# Functional Phenotypic Diversity of Regulatory T Cells Remaining in Inflamed Skin

**DOI:** 10.3389/fimmu.2019.01098

**Published:** 2019-05-17

**Authors:** Ryoyo Ikebuchi, Maika Fujimoto, Yasutaka Nakanishi, Hiromi Okuyama, Taiki Moriya, Yutaka Kusumoto, Michio Tomura

**Affiliations:** ^1^Laboratory of Immunology, Faculty of Pharmacy, Osaka Ohtani University, Tondabayashi, Japan; ^2^Research Fellow of Japan Society for the Promotion of Science, Tokyo, Japan

**Keywords:** regulatory T cells, diversity, contact hypersensitivity response, skin, single cells

## Abstract

Regulatory T cells (Tregs) migrate between lymphoid and peripheral tissues for maintaining immune homeostasis. Tissue-specific function and functional heterogeneity of Tregs have been suggested, however, correlation between them and inter-tissue movement remain unknown. We used a contact hypersensitivity model of mice expressing a photoconvertible protein for tracking migratory cells. After marking cells in skin, we purified Tregs exhibiting a different migration pattern [Tregs recruiting to or remaining in the skin and emigrating from the skin to draining lymph nodes (dLNs) within half a day] and examined single-cell gene and protein expression profiles. Correlation and unsupervised clustering analyses revealed that Tregs in both skin and dLNs comprised two subpopulations, one highly expressing Nrp1 with variable CD25, Granzyme B, and/or CTLA-4 expression and another with 3 subsets strongly expressing CD25, Granzyme B, or CTLA-4 together with CD39. Characteristic subsets of Tregs remaining in the skin displayed higher CD25 and CD39 expression and lower Granzyme B and CTLA-4 expression compared with Tregs migrating to the skin. In addition, CCR5 expression in Tregs in skin was positively and negatively correlated with CD39 and Nrp-1 expression, respectively. To assess the predictive value of these data for immunotherapy, we blocked CCR5 signaling and found modest downregulation of CD39 and modest upregulation of Nrp1 expression in skin Tregs. Our data reveal a high functional diversity of Tregs in skin that is strongly related to trafficking behavior, particularly skin retention. Modulation of tissue-specific trafficking and function is a promising clinical strategy against autoimmune, infectious, and neoplastic diseases.

**Significance Statement:**

Regulatory T cells (Tregs) are essential for maintaining immune homeostasis. To reveal tissue-specific immunoinhibitory functions and inter-tissue movement correlation based on Treg functional heterogeneity, we examined single-cell gene and protein expression profiles of Tregs recruited to, remaining in, or emigrating from the contact hypersensitivity-induced inflamed skin. Tregs in skin were composed of several subpopulations; one with high Nrp1 expression and another with 3 subsets strongly expressing CD25, Granzyme B, or CTLA-4 together with CD39. Tregs remaining in skin displayed highCD25, CD39, and CCR5 expression, and CCR5 signaling blockade downregulated CD39. A high Treg functional diversity in skin is strongly related to trafficking behavior. Tissue-specific trafficking and functional modulation are a promising clinical strategy against autoimmune, infectious, and neoplastic diseases.

## Introduction

Regulatory T cells (Tregs) mediate antigen-specific suppression of immune reactions in inflamed tissues (1). Soluble factors and cell surface molecules expressed by Tregs contribute to distinct immunosuppressive pathways (2, 3). For example, the immunoinhibitory cytokines interleukin (IL)-10 and TGF-β released by Tregs directly modulate conventional T cells (Tconvs) (2), while the ecto-enzymes CD39 and CD73 produce adenosine, which inhibits Tconv activation and survival (4). The IL-2 receptor α chain (CD25) on the Treg surface indirectly inhibits Tconv expansion by binding IL-2 (3), an important cytokine for Tconv proliferation. Granzyme B (GzmB) and perforin induce cytolysis of effector cells (5, 6). Alternatively, cell surface molecules CTLA-4, LAG-3, and neuropilin-1 (Nrp1) impair dendritic cell (DC)-mediated Tconv activation (2, 3). Specifically, CTLA-4 and LAG-3 outcompete CD28 and T cell receptor expressed on Tconvs for binding to CD80/86 and MHC class II on DCs, and Nrp1 stabilizes DC–Treg contact, thereby preventing antigen presentation to Tconvs.

Several studies using single-cell expression analysis have revealed heterogeneity of cells among tumor cells and epidermal cells (7, 8). Human Treg phenotype is also heterogeneous, and only some Tregs, for example express IL-10 and CD39 (9, 10). Our previous report using single-cell analysis also revealed expression heterogeneity of immune function-related genes by mouse Tregs isolated from draining lymph nodes (dLNs) of contact hypersensitivity (CHS)-induced inflamed skin (11). Almost all Tregs express CTLA-4, TGF-β, or both, however, some Tregs only express GzmB. In addition, rare subset of Tregs express IL-10 and almost all of them co-express all three molecules; CTLA-4, TGF-β and GzmB (11). These reports suggest substantial functional diversity among Tregs; however, expression heterogeneity of other functional molecules is currently uncertain.

Migration and tissue tropism of Tregs are well-studied due to specific chemokine receptor profiles in different tissues (12–14); furthermore, Campbell et al. delineated the relationships between immunosuppressive mechanisms and tissue tropism for Tregs. Tregs target DCs in lymphoid tissues for immunoregulation driven by CTLA-4, while IL-10 is essential for immunoregulation in non-lymphoid tissues, such as mucosa and skin (15). We also demonstrated that local immune inhibition is influenced by Treg's capacity to remain in the tissue (11), defined as the proportions of cells remaining in a tissue during a certain period of time, such as half a day, and measured using mice models expressing a photoconvertible protein (16, 17). We also found Tregs highly expressing both GzmB and IL-10 in dLNs; they were subdivided into two subsets with high and low skin-remaining capacity, and only one subset with high skin-remaining capacity suppressed CHS response after cell transfer to inflamed skin (11). This suggests that high functional capacity alone is not sufficient, and that tissue tropism or tissue-remaining capacity is also required for inhibition of local immune response by Tregs.

Based on these reports, we hypothesized that Tregs remaining in peripheral tissue, cells remaining for half a day in already inflamed skin in this study, have unique functional diversity. To test this hypothesis, we examined single-cell expression profiles of Tregs remaining in inflamed skin tissue by single-cell real-time PCR array (scqPCR) and full-spectral flow cytometry (FCM) (18) with analysis using an unsupervised clustering method. In addition, we modulated the functional phenotypic diversity of skin Tregs by inhibiting migration signaling. These observations revealed multiple Treg expression phenotypes in skin with distinct migratory behaviors, findings that may provide experimental targets for further understanding tissue-specific Treg function and therapeutic targets to modulate peripheral Tregs.

## Materials and Methods

### Mice

KikGR knock-in mice (19) were crossed with Foxp3^hCD2/hCD52^ mice (20) in which Foxp3^+^ cells express human CD2 (hCD2) and human CD52 fusion protein on the cell surface. Foxp3^hCD2/hCD52^ mice were kindly provided by Dr. Shohei Hori, IMS, RIKEN, Japan. Mice were maintained in specific pathogen-free facilities at Osaka Ohtani University. All animals were treated according to the Guidelines for Proper Conduct of Animal Experiments (Science Council of Japan), and all protocols were approved by the Institutional Animal Care and Use Committee of Kyoto University Faculty of Medicine and the Animal Research Committee of Osaka Ohtani University.

### Contact hypersensitivity (CHS)

Contact hypersensitivity was induced by topical application of 2,4-dinitro-1-fluoro-benzene (DNFB; Nacalai Tesque) as previously described (11). Briefly, mice were sensitized by application of 0.75% DNFB to the shaved lower abdominal skin, and 5 days later were challenged with 0.45% DNFB to the skin of each ear. Skin cells at the inflammation site were photoconverted 2.5 days following DNFB challenge as described previously (19). Briefly, the mice were anesthetized, and the ear skin was subjected to 2 min exposure to violet light (436 nm, 100 mW/cm^2^) using SP500 spot UV curing equipment with a 436 nm band-pass filter (USHIO). The exposure converts KikGR from green (KikGR-Green) to red (KikGR-Red). This light exposure protocol alone does not induce significant inflammation of the skin (16, 21). Skin and dLN cells were analyzed the following day.

### Cell Isolation

After removal of fat and cartilage tissues, ear skin was incubated for 45 min in RPMI 1640-medium (Sigma Aldrich) containing 10% fetal bovine serum (HyClone), collagenase II (Worthington Biochemical), and DNase I (Calbiochem) at room temperature. After mincing with scissors, the skin was incubated in the same solution for 25 min at 37°C. After adding 0.5 M EDTA, cell suspensions were filtered through a 70-μm cell strainer (BD Biosciences). dLN cells were also treated with the enzymes as described above.

### Antibodies and Flow Cytometry (FCM)

Single-cell suspensions of skin and dLN cells were incubated with anti-CD16/CD32 (TONBO Biosciences) for 5 min, and then stained using the fluorochrome-conjugated antibodies described in Table S1. Intracellular GzmB and CTLA-4 were stained using Fixation Buffer and 10 × Permeabilization Wash Buffer (BioLegend) as described in the manufacturer's protocol. The fluorescence intensity of KikGR-Red was reduced by fixation, thus, the proportion of KikGR-Red^+^ cells was lower in samples subjected to intracellular staining than in fresh samples. Dead cells were stained with 7-AAD (eBioscience) or propidium iodide (Wako) and excluded from our analyses. Stained samples were analyzed using a SP6800 Spectral Cell Analyzer (SONY). All data were exported as FCS files and analyzed using FlowJo software (Tree Star).

### Measurement of Single-Cell Gene Expression

Tregs were sorted from the pooled inflamed skin and dLN samples of 5 mice using a Moflo Astrios flow cytometer (Beckman Coulter). The capture of individual Tregs, cell lysis, RT-PCR, and pre-amplification of 96 genes were performed using a C1 Single-Cell Auto Prep system (Fluidigm) with a Single Cell-to-CT kit (Life Technologies) and C1 Single-Cell AutoPrep Reagent kit (Fluidigm) as described in the manufacturer's protocol. Amplicons from single Tregs were collected and stored at −30°C. scqPCR was performed using 96.96 Dynamic Arrays (Fluidigm) and a BioMark system (Fluidigm) as described in the manufacturer's protocol. The primers used in the C1 and BioMark are listed in Table S2. To reduce non-specific amplification, nested primer pairs were used for amplification of nine genes by scqPCR.

### Data Analysis of Single-Cell Expression Profiles

Single-cell expression values were derived from qPCR data by the Ct method using Real-Time PCR Analysis software (Fluidigm) following exclusion of failed reactions. To ensure that samples represented healthy Tregs, we included only data of single cells expressing all reference genes (*Actb, B2m, Gapdh*, C*d3e*, and *Cd4*) in subsequent analyses.

The FCM data obtained by SP6800 were compensated using a spectral unmixing algorithm (18), and proper compensation was confirmed using SP6800 software. To reduce the possibility that inappropriate compensation generated the correlations among expression levels, we obtained several FCM data sets with two color panels for correlation analysis. Single-cell protein expression data of Treg subpopulations were exported as CSV files by FlowJo software following conventional FCM gating analysis. All Tregs were identified as CD45^+^ CD4^+^ hCD2^+^ cells.

All analyses and data visualization were performed using R 3.1.2 (the R Foundation for Statistical Computing). For generating volcano plots, data were compared via Student's *t*-test. For clustering analysis, we used the Manhattan, Euclidean distance, group average, and Ward's method and chose a proper combination to distinguish objective clusters (see **Figures 2, 5**, Figures S1, S2). For multiple comparisons of clusters, we used the Fisher's exact test with Benjamini-Hochberg correction. For correlation analysis, Spearman's rank correlation coefficients were calculated because the data values did not follow a Gaussian distribution. In principal component analysis (PCA), the number of significant PCs was determined by parallel analysis. Text plots were used to illustrate the relationships between molecule expressions following PCA. Analysis by t-distributed stochastic linear embedding (tSNE) was also used to identify Treg subsets (22). These analyses were run using a custom R script based on the “heatmap.2” function of the “gplots” package, “pairwise.fisher.test” of “fmsb,” and “prcomp” function. When running tSNE with different perplexity, we used “Rtsne” of “Rtsne” (theta = 0.2, max_iter = 1,000), and in other cases, we used “tsne” of “tsne” (perplexity = 30, max_iter = 500). The “sample_n” function of the “dplyr” package was used to generate pooled data, including equivalent events of replicated data, resulting in partial normalization of the contribution of each animal. We did not transform FCM data before applying tSNE. Clustering of individual cells in tSNE plots was performed based on Phenograph (23) using two tSNE dimensions. Phenograph was run using “cytofkit” packages. The Fisher's exact test was performed to test statistical significance in the difference in the number of KikGR-Red^+^ and KikGR-Red^−^ cells in each cluster.

### Reagents and Treatment

Eight micrograms of DAPTA (TOCRIS), a specific antagonist of CCR5 (24–26), was injected subcutaneously into the ventral surface of the ears 2.5 days following DNFB challenge. Immediately after DAPTA treatment, ear cells were photoconverted by violet light as described above. The phenotypes of ear skin cells were analyzed the following day.

### Statistics

The paired *t*-test and Wilcoxon's matched pair test were performed after checking data for normality using the D'Agostino and Pearson normality test. All tests were conducted using GraphPad Prism version 5.0 and 7.04 (GraphPad Software). Data in bar graphs and scatter dot plots are presented as mean ± SEM. A *P* < 0.05 was considered statistically significant for all tests.

## Results

### Functional Phenotypic Diversity of Tregs in Contact Hypersensitivity Model Mice

To distinguish among Tregs migrating to inflamed skin, remaining in inflamed skin, and emigrating from inflamed skin to dLNs, we established a CHS model in mice expressing a photoconvertible protein, KikGR, that shifts from green (KikGR-Green) to red (KikGR-Red) in response to violet light exposure (19). Local exposure at the CHS site to violet light thus allows for the tracking of Tregs emigrating to dLNs in the days following exposure.

**Figure 1:**
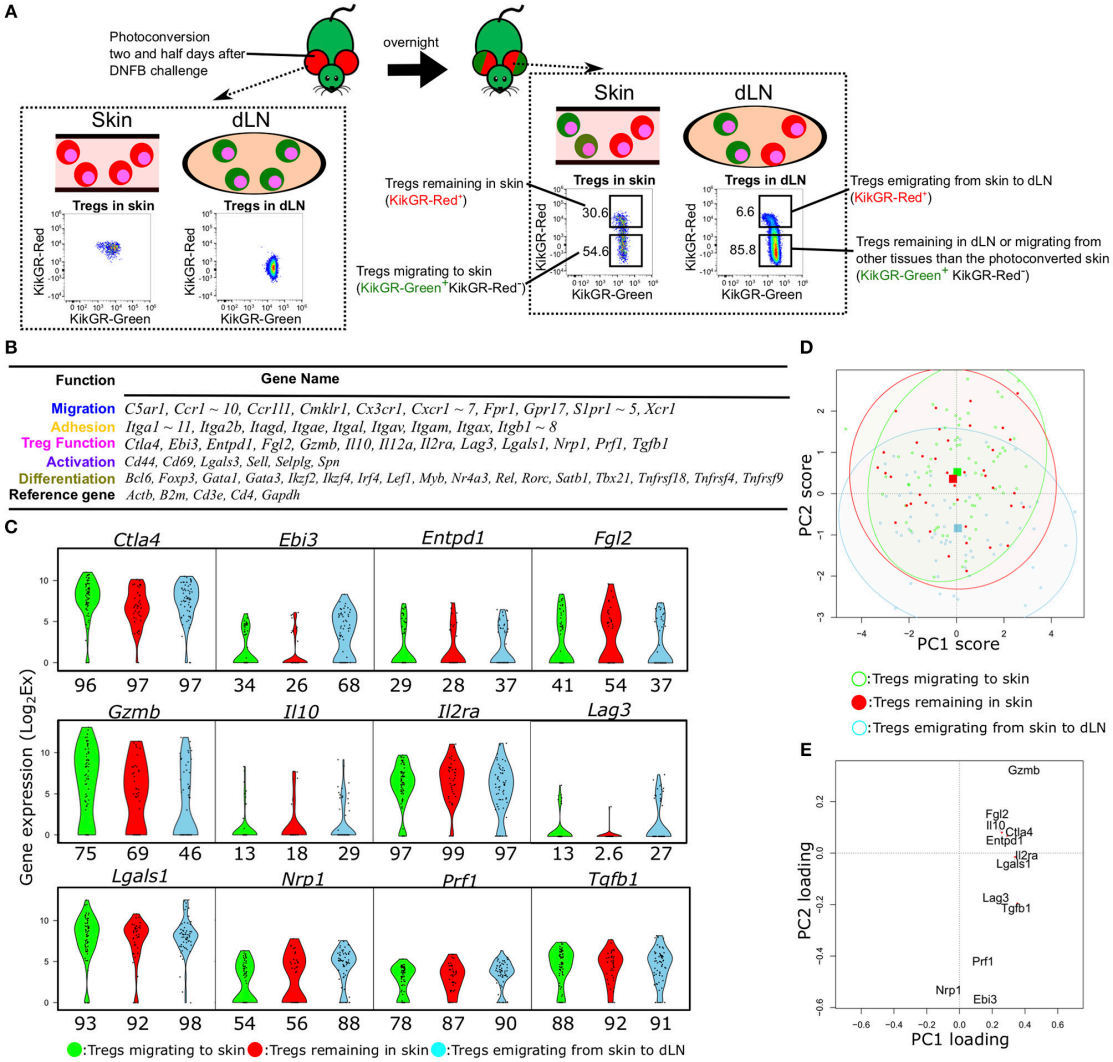
Single-cell qPCR analysis of Tregs migrating to, remaining in, or emigrating from CHS-induced inflamed skin. **(A)** Experimental scheme for discrimination of Tregs migrating to, remaining in, emigrating from the inflamed skin and FCM gating for KikGR-Green^+^ (KikGR-Red^−^) or KikGR-Red^+^ cells. Values in plots indicate the percentage of the parent population. Data are representative of four independent experiments with two mice per group. **(B)** Genes measured by scqPCR. Genes are grouped by their representative function. **(C)** Violin plots showing expression levels of genes related with Treg function. Percentages under each plot indicate the proportion of cells that expressed the gene. **(D)** Principal component (PC) projections of individual Tregs within the three migratory populations. PC2 and PC3 are shown (accounting for 12.5 and 10.4% of the total variation, respectively). The confidence ellipses help to visually compare relationships among the populations. Squares indicate the mean value for each subset with the same color. **(E)** PC projection of 12 genes, showing contributions to PC1 and PC2.

Ear skin cells were photoconverted two and half days following CHS induction by local DNFB challenge. The photoconversion changed fluorescence of skin cells, including Tregs, to KikGR-Red but did not immediately influence the fluorescence of dLN Tregs (Figure 1A left). The next day, however, cells migrating to or remaining in skin were identified as KikGR-Green (Red^−^) or KikGR-Red^+^ (50.0 ± 3.3% or 31.5 ± 0.9%, mean ± SEM, *n* = 8), respectively, and those emigrating to dLN from inflamed skin as KikGR-Red^+^ (5.0 ± 0.4%. mean ± SEM, *n* = 8, Figure 1A right).

For each Treg population, we measured the single-cell expression levels of 96 transcripts encoding molecules associated with migration, adhesion, Treg function, cell activation, and differentiation (Figure 1B). Fifteen genes were not detected in any cells of the three Treg migratory populations or amplified by non-specific PCR reactions. Consequently, we obtained expression data for 81 genes from 68 individual cells migrating to inflamed skin (KikGR-Red^−^ in skin), 39 remaining in skin (KikGR-Red^+^ in skin), and 59 emigrating from inflamed skin to dLNs (KikGR-Red^+^ in dLN) after exclusion of PCR data with no expression of the five reference genes *Actb, B2m*, C*d3e, Cd4*, and *Gapdh*.

Hierarchical clustering analyses yielded a unique cell cluster mainly consisting of Tregs migrating to skin (Figure S1A). When dividing individual cells into eight clusters, Tregs migrating to skin were included in clusters 7 and 8 more than in other clusters (*p* < 0.05, Figure S1A). *Itga11, Myb*, and several Treg function-related genes, including *Gzmb, Lgals1*, and *Ctla4*, were identified as a gene set highly expressed by the Tregs migrating to skin in clusters 7 and 8 compared with other cells (Figure S1B). These four genes except *Ctla4*, were not significantly altered in comparison of total Tregs migrating to skin with those emigrating from skin to dLNs and those remaining in skin (Figures S1C,D). Several adhesion-, migration-, and differentiation-related genes were upregulated two-fold or more in Tregs emigrating from skin to dLNs relative to the other two subsets (Figures S1C,E). Three genes (*Ctla4, Tnfrsf4*, and *Tnfrsf18*) were more highly expressed in Tregs migrating to skin than in Tregs remaining in skin (Figure S1D), while no genes were upregulated in Tregs remaining in skin compared to the other populations (Figures S1D,E). These data suggest that the three Treg populations differ only slightly in their expression profiles at the group level but may exhibit functional heterogeneity at the single-cell level. Thus, single-cell analyses are required to compare the functional diversity among these three Treg populations.

We next focused on the difference in expression of functional molecules to evaluate functional phenotypic diversity of the three Treg populations. The genes *Ctla4, Il2ra, Lgals1, Prf1*, and *Tgfb1* were expressed in more than 80% of Tregs within the three populations (Figure 1C), while *Il10*- and *Lag3*-expressing Tregs were rare among the skin Tregs (both KikGR-Red^−^ and KikGR-Red^+^) and *Il12a* was not detected in any Tregs (Figure S1A). To examine the underlying single-cell diversity, we performed PCA using expression data of 12 Treg function-related molecules. PCA projection of the first dimension of PCA (PC1) and PC2, accounting for 26.5 and 12.5% of the total variation, showed a great degree of diversity among individual Tregs (Figure 1D), although it did not contribute to distinguish between the three populations at all. They were extensively plotted, suggesting single-cell diversity, and we were able to identify the key features generating this diversity because PC1 and PC2 consisted of contribution from 12 function-related molecules (Figure 1E). *Gzmb* had the highest PC1 and PC2 scores both, and *Nrp1* did the lowest PC1 score and the second lowest PC2 score, suggesting that the most contribution to generation of this diversity. PCA projection of PC2 and PC3 (10.4% of the total variation) also show the diversity to some extent, but did not show clear sub-clusters within each migration population (Figures S1F,G). *Lgals1* and *Il2ra* or *Il10* and *Lag3* were main contributors for positive or negative PC3 score (Figure S1G).

### Nrp1-expresing Skin Tregs and CD25-, CD39-, CTLA-4-, or GzmB-expressing Skin Tregs Form Distinct Subsets

**Figure 2:**
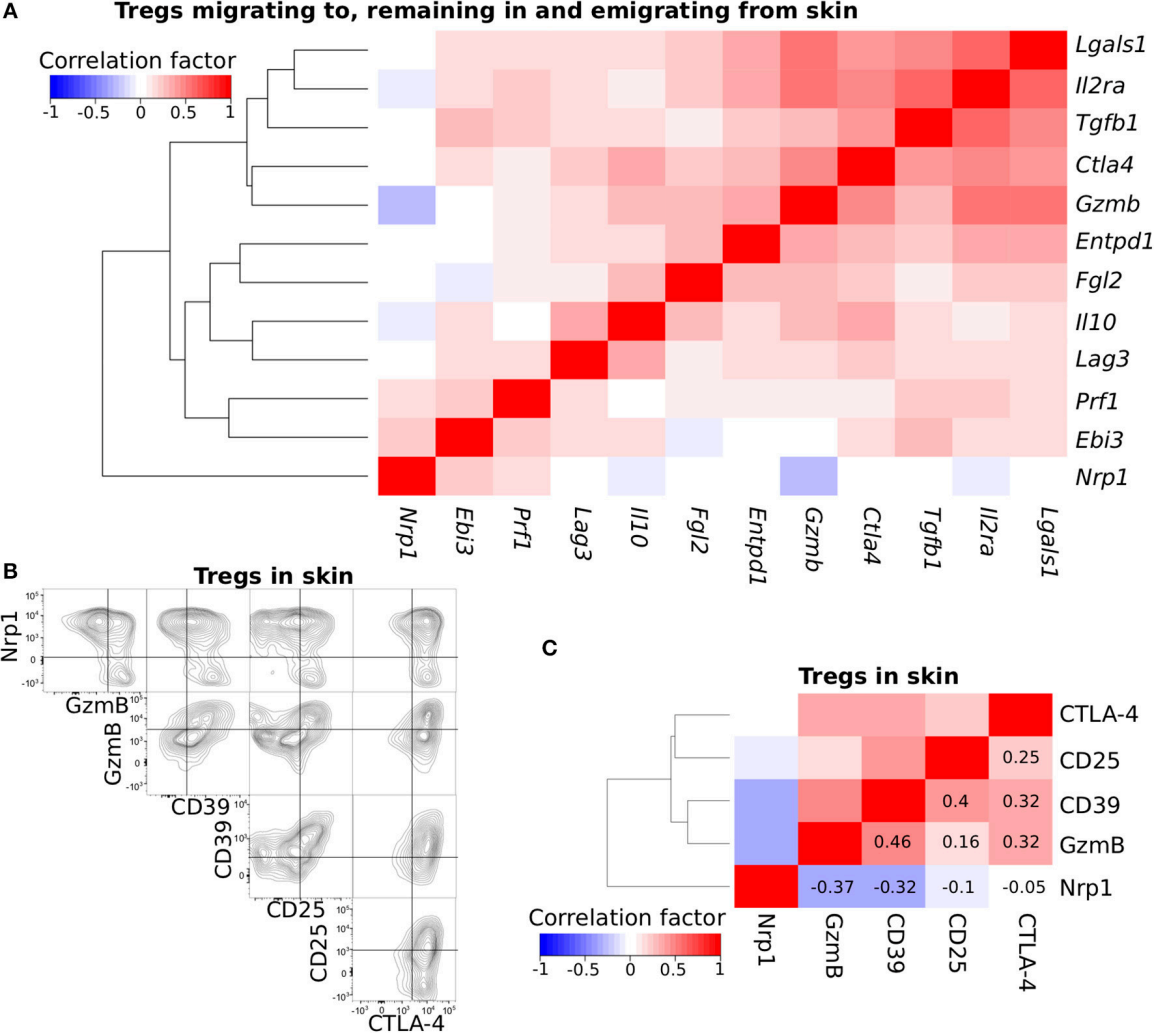
Cross-correlation analysis of functional molecule expression profiles. **(A)** Cross-correlation analysis of the expression profiles for genes related to Treg function. scqPCR data was formed by concatenating the data from the three Treg populations. Colors indicate positive (red) or negative (blue) correlations. **(B)** Nrp1, GzmB, CD39, CD25, and CTLA-4 protein expression levels of Tregs in inflamed skin. Flow cytometry (FCM) data are representative of three independent experiments. Lines in plots indicate boundaries between positive and negative cells. **(C)** Cross-correlation analysis of the expression profiles for five proteins using pooled FCM data (*n* = 3). Clustering was performed by the Manhattan distance and group average method.

Expression data from hundreds or thousands of single cells were required for detailed analysis of Treg functional diversity. Flow cytometry can obtain protein expression data for thousands of single cells; however, we were not able to obtain single-cell protein expression data for the 12 function-related molecules due to limited spectral resolution of fluorescence detectors and available antibodies. To narrow down the molecules generating functional diversity of skin Tregs, we examined the correlations among the 12 genes related to Treg function (Figure 2A). To do so, we pooled the scqPCR data obtained from the three Treg migration populations because they exhibited similar expression profiles. *Nrp1* was not clustered with the others consistent with the lowest PC1 score in PCA (Figure 1E), and its expression was inversely correlated with *Il2ra* (encoding CD25), *Gzmb* (encoding GzmB), and *Il10* (Figure 2A). Five genes, *Lgals1, Il2ra, Tgfb1, Ctla4*, and *Gzmb*, were grouped in one distinct cluster and other genes including *Entpd1* (encoding CD39), *Il10, Prf1* and so on in another distinct cluster.

To confirm the correlations observed using scqPCR data, we conducted FCM analysis on five proteins, CD25, CTLA-4, GzmB, CD39, and Nrp1, each of which was included in different clusters (Figure 2A) and some of which had high or low scores of PC1, 2, and 3 (Figure 1E, Figure S1G). Each was stained using five specific fluorescence-conjugated antibodies (Figure S2A) in individual skin Tregs and enumerated by FCM (Figure 2B). Most Nrp1^−^ skin Tregs strongly expressed GzmB and CD25 (consistent with scqPCR; Figure 2A) or CD39 and CTLA-4, while Nrp1^+^ skin Tregs expressed these 4 proteins heterogeneously. CD39 expression was positively correlated with each of GzmB, CD25, and CTLA-4 expression. A dendrogram also showed these correlations and generated two major clusters, one with Nrp1 alone and the other with GzmB, CD39, CD25, and CTLA-4 (Figure 2C). To exclude the possibility that inappropriate levels of fluorescence compensation generated these correlations, we measured expression levels of all 5 proteins by staining Tregs with another set of five fluorescence-conjugated antibodies (Figure S2B). This analysis yielded the same correlation pattern, suggesting that heterogeneous expression levels of these five molecules contribute to skin Treg functional diversity at the single-cell level.

### High Functional Phenotypic Diversity of Tregs Migrating to, Remaining in, and Emigrating From Inflamed Skin

**Figure 3:**
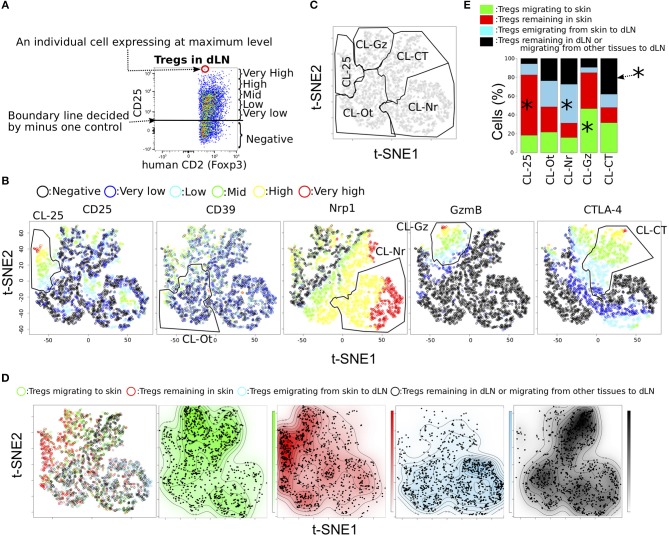
Functional phenotypic diversity of skin and dLN Tregs with different migration activities. **(A)** Example defining the six expression levels of effecter molecules in Tregs. **(B,C)** tSNE projections for five proteins expressed in Tregs for pooled data from individual Tregs migrating to and remaining in skin and from Tregs emigrating from skin to dLN and Tregs remaining in dLN or migrating from other tissues to dLN (*n* = 3). Six expression levels (defined as in a) are indicated by different colors. Clusters named CL-25, CL-Nr, CL-Gz, CL-Ot, and CL-CT were defined by manual gating (polygons). **(D)** Migration activities of individual cells are indicated in the tSNE plots by different colors. Distribution of the cells within each population is indicated by the four contour plots. **(E)** Proportions of Treg subpopulations with different migration activities within each cluster defined by manual gating. Asterisks indicate most significant proportions in each cluster.

CTLA-4 and Nrp1 mainly contribute to suppression of inflammatory pathways mediated by dendritic cells (DCs), such as antigen presentation, by blocking co-stimulation molecules and stabilizing the interaction of DCs with Tregs (15, 27). We thus hypothesized that Tregs in dLNs would consist mainly of cells with high expression of CTLA-4 and/or Nrp1, while Tregs remaining in skin would not. To address differences in CTLA-4 and Nrp1 expression between within each migration population, we visualized the functional phenotypic diversity and migratory activity of Tregs in a single plot by unsupervised tSNE analysis, a dimensionality reduction method (22, 28) that maintains the overall data structure at single-cell resolution and shows improved segregation of subpopulations compared to PCA. We first concatenated equal numbers of cells (*n* = 1,193) from each Treg population (migrating to, remaining in, and emigrating from skin) and the KikGR-Red^−^ dLN Tregs resident in dLN during inflammation or migrated from tissues other than the photoconverted skin (Figure 1A). These tSNE analyses were performed based on expression levels of CD25, CD39, Nrp1, GzmB, and CTLA-4. Expression intensities in the tSNE plots were displayed at six levels defined by a traditional FCM gating strategy (Figure 3A). For example, each CD25^+^ Tregs were divided into five subsets (very low, low, middle, high, and very high) based on expression level between the highest expression intensity and the boundary line between positive and negative decided by minus one control. The tSNE projection showed several clusters that were segregated based on a combination of CD25, Nrp1, GzmB, and CTLA-4 expression levels (Figure 3B), and we manually gated five clusters (Figure 3C). For example, almost all cells in cluster CL-25 or CL-CT expressed CD25 or CTLA-4 more highly than low levels and did not express GzmB, and CL-Gz expressed GzmB at higher than middle levels and included many Nrp1^−^ cells, consistent with correlation analysis (Figure 2C). CL-Nr and CL-Ot (others) were clustered based on both positional information and Nrp1 expression level. CL-Nr expressed Nrp1 at high and very high levels and CTLA-4 at low, very low, and negative levels. These clusters also held the tSNE projections with different perplexities; from 30 to 500 (Figure S3).

To assess whether the cell clusters generated by tSNE based on functional molecules have different migratory activities, we visualized the distribution of the four Treg migratory populations in the tSNE contour plot, those migrating to the CHS skin (Green in Figure 3D), remaining in the CHS skin (Red), emigrated from CHS skin (Blue), and remaining in dLN during CHS or migrating from other tissues to dLN (Black). This analysis provided a general discrimination of these populations according to functional protein expression level (Figure 3E and Table S3). For example, CL-Ot homogeneously included each population with different migratory activity. CL-25 and CL-Gz mainly consisted of skin Tregs (Tregs migrating to and remaining in skin). Tregs remaining in skin were most common in CL-25 cluster (259/405 cells), whereas approximately half CL-Gz were Tregs migrating to skin (268/573 cells). CL-Nr cluster that strongly expressed Nrp1 but did not express CTLA-4 mainly consisted of dLN Tregs, Tregs emigrating from skin to dLNs and Tregs remaining in dLNs (645 and 430 per 1,562 cells). Most Tregs remaining in dLNs or migrating from skin to dLN expressed CTLA-4 or Nrp1 at high levels (CL-CT or CL-Nr cluster), consistent with the hypothesis that CTLA-4^+^ and Nrp1^+^ Tregs possess tissue tropism for dLNs rather than skin. A wide distribution of Treg expression phenotypes was observed in the population migrating to skin, including not only CL-Gz but also fractions of the CL-CT and CL-Nr clusters, consisted dLN Tregs more than skin Tregs (Figures 3C,E and Table S3), suggesting that Tregs migrating to skin have characteristics of both Tregs remaining in skin and Tregs in dLNs. Meanwhile, Tregs remaining in skin most frequently were included in CL-Ot, followed by CL-25 (Table S3). This observation revealed that each Treg population exhibiting a different migration pattern possesses a unique expression phenotype of functional molecules.

### t-SNE and Phenograph Analyses Identify Unique Functional Phenotypes Among Treg Subsets Remaining in Inflamed Skin

In previous reports, we found that several tens of thousands of Tregs migrated from the CHS-induce inflamed skin to dLNs over the day following induction (21) and speculated that the principle function of those remaining in the inflamed skin is to suppress inflammation (11). It is therefore of particular clinical significance to investigate the expression profiles of Tregs remaining in skin.

**Figure 4:**
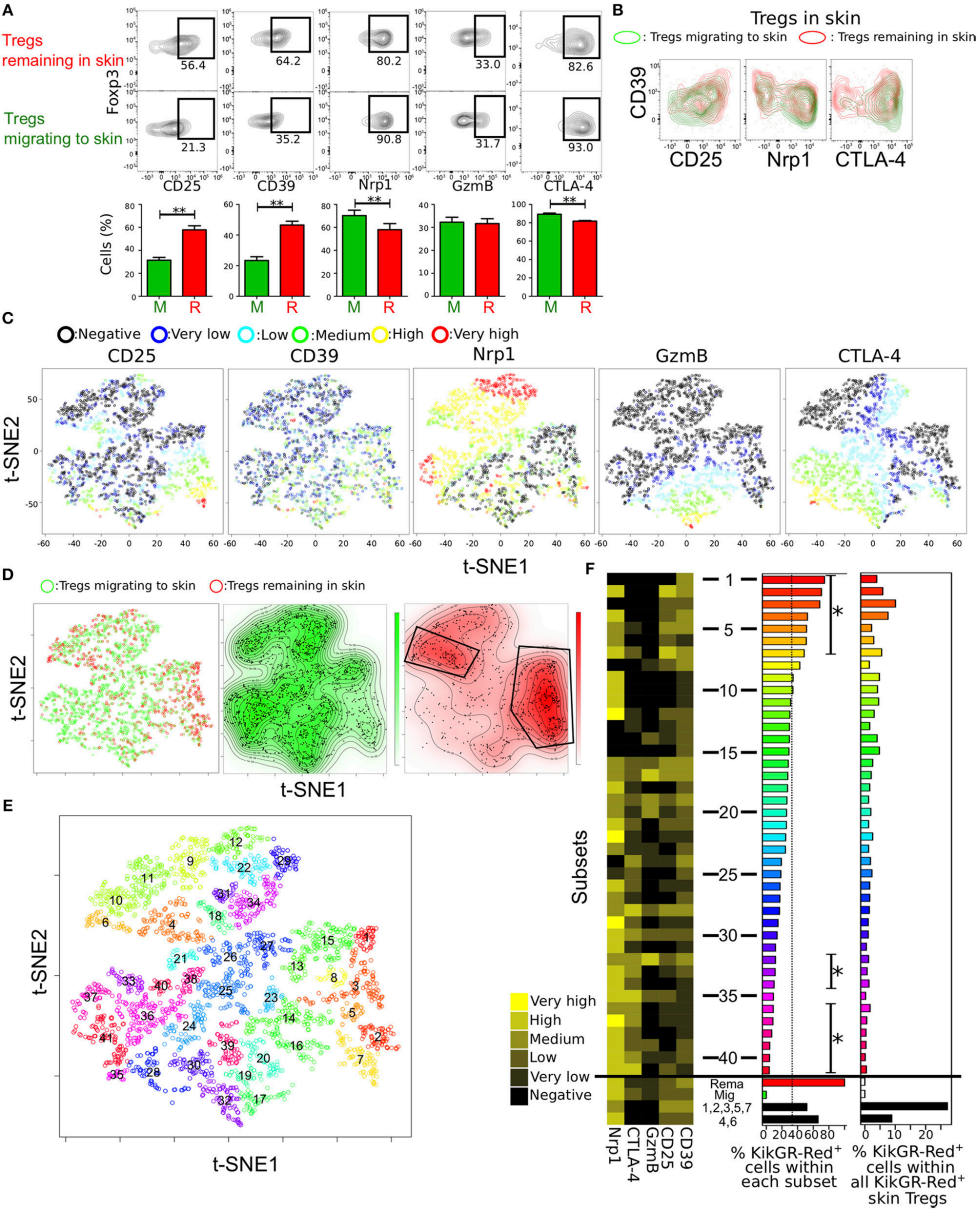
Identification of distinct Treg subsets remaining in skin by automated clustering analysis of expression data. **(A)** Proportions of cells expressing each of five molecules related to Treg function in Tregs migrating to and remaining in skin (*n* = 19, 23, 18, 12, and 8 for CD25, CD39, Nrp1, GzmB, and CTLA-4 expression, respectively). **(B)** Contour plots showing CD39 expression and each of CD25, Nrp1, and CTLA-4 expression in Tregs migrating to and remaining in skin. FCM data are representative of three independent experiments. **(C,D)** tSNE projections of the five proteins expressed by Tregs from pooled data of individual cells migrating to and remaining in skin (*n* = 3). Expression levels and migration activity of individual cells are indicated as in Figure 3. **(E)** Treg subsets identified by automated clustering analysis using expression data of the five molecules and two dimensions of tSNE. Numbers indicate center points of the subsets. **(F)** Mean expression of the five molecules (heatmap in left) and proportion of KikGR-Red^+^ cells (Tregs remaining in skin; bar plots in the center) in each of forty-one subsets identified in **(E)**. Tregs remaining in and migrating to skin and two subsets consisted of cells in clusters 1, 3, 4, and 7 and clusters 2 and 6. Percentages of KikGR-Red^+^ cells within each cluster shown in bar plots at left. Asterisks indicate significantly more or less KikGR-Red^+^ cell numbers than KikGR-Red^−^ cell numbers in each cluster. Percentages of KikGR-Red^+^ cells in each cluster within all KikGR-Red^+^ skin Tregs are shown in bar plots at right.

We first compared the proportions of each functional molecule-expressing subset between populations remaining in skin and migrating to skin (Figure 4A). Tregs remaining in skin expressed CD25 and CD39 at higher levels and Nrp1 and CTLA-4 at lower levels than Tregs migrating to skin. This population analysis revealed substantial differences in protein expression profiles between these two Treg populations. However, traditional FCM gating did not clearly distinguish the two populations (Figure 4B) or identify unique phenotypes among Tregs remaining in skin. To overcome this problem, we performed tSNE analysis on 2,994 events including actual proportions of Tregs migrating to and remaining in skin (Figures 4C–E). Pooled data from triplicate analyses was used for this tSNE analysis and we confirmed the absence of bias due to experimental replicates (Figure S4A). We observed gradient expression of CD25, Nrp1, GzmB, and CTLA-4 in tSNE projections with different perplexity (Figure S4B) as same as Figure 3B. CD39 was expressed highly in cells with high expression of CD25 and GzmB, suggesting that CD39 expression is a strong indicator of CD25, GzmB, and CTLA-4 expression levels, consistent with correlation analysis (Figure 2C).

Two distinctive clusters were identified in Tregs remaining in skin by manual gating (Figure 4D). To functionally distinguish the two clusters in the Treg population remaining in skin, unsupervised cluster analyses were performed using Phenograph (23), which showed good clustering performances of FCM data than other methods (29). We used different parameters, such as *k* = 18, 12, and 30 (number of nearest neighbors) in Phenograph using two dimensions of tSNE analysis (Figures 4E,F, Figures S5A,B). Clusters generated by the algorithm were labeled “1” for maximum number of subsets in order of the KikGR-Red^+^ cell proportion (highest to lowest).

Clusters of small numbers (skin remaining cells-concentrated clusters), such as clusters 1, 2, 3, 5 and 7 or 4, 6, 9, 10, and 11 were in right lower or upper region of the plot, in which CD25^mid/high/veryhigh^ cells or CTLA-4^−^ Nrp1^mid/high^ cells were located, as shown in Figure 4D. GzmB^mid/high/veryhigh^ cells were included in clusters of heterogeneous numbers (17, 19, 20, 30, 32, and 39) and CTLA-4^mid/high/veryhigh^ cells in many clusters of high numbers, suggesting low proportion of KikGR-Red^+^ cells. We next visualized expression patterns of the five functional molecules, proportions of KikGR-Red^+^ cells within each cluster and percentages of KikGR-Red^+^ cell number in each cluster relative to all KikGR-Red^+^ skin Tregs (Figure 4F). Clusters from 1–7 or clusters 32, 33, 34, 36, 37, 38, 39, 40, and 41 significantly consisted of more or less KikGR-Red^+^ cells compared with KikGR^−^ cells (left bar plot in Figure 4F). Skin remaining cells-concentrated clusters 1, 2, 3, 4, and 6, and clusters 9, 10, 11, 15 also, expressed both CTLA-4 and GzmB at undetectable levels. Clusters with Nrp1 expression at undetectable levels or both CTLA-4 and GzmB expression at undetectable levels were frequently observed in upper half of clusters (clusters 1–20) compared with the lower half (clusters 21–41, *p* = 0.043 or 0.0005). We observed similar cluster distribution in the different clustering patterns (*k* = 12 and 30, Figures S5A,B). To identify expression profiles of the two clusters within the skin-remaining Treg population identified by manual gating (Figure 4D), we calculated average expression levels in the concatenated cluster set including 1, 2, 3, 5, and 7 as well as the set including 4 and 6 (Figure 4F). Both cluster sets contained large proportions of CTLA-4^−^ GzmB^−^ cells and expressed CD25 and CD39 at more than low levels. Therefore, our analysis reveals functional diversity of characteristic subsets within the population of Tregs remaining in inflamed skin. Migration activity of Tregs in inflamed skin may be explained by functional phenotypic diversity.

### Functional Phenotypic Diversity of Skin Tregs Is Altered by Inhibiting Migration Signaling

**Figure 5:**
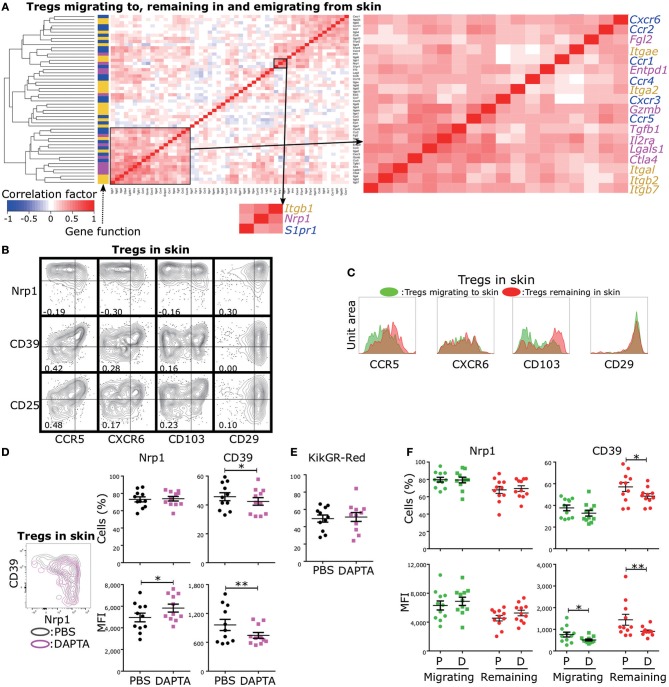
Identification of migration-related molecules influencing functional phenotypic diversity of skin Tregs. **(A)** Cross-correlation analysis of the expression profiles of genes related to Treg function (magenta), migration (blue), and adhesion (yellow). scqPCR data were formed by concatenating the data of the three Treg populations (Tregs migrating to, remaining in, and emigrating from skin). Colors in cells indicate positive (red) or negative (blue) correlations. Clustering was performed by the Manhattan distance and group average method. **(B)** Nrp1, CD39, CD25, CD29, CD103, CXCR6, and CCR5 protein expression by Tregs in skin. FCM data are representative of three independent experiments. Lines in plots indicate boundaries between positive and negative cells. **(C)** Replicative data of CD29, CD103, CXCR6, and CCR5 protein expression by Tregs migrating to and remaining in skin (*n* = 3). **(D,E)** Nrp1 and CD39 expression **(D)** and Proportions of KikGR-Red^+^ cells **(E)** in skin Tregs of mice treated with PBS (control) or DAPTA. **(F)** Nrp1 and CD39 expression by Tregs migrating to and remaining in skin of mice treated with PBS or DAPTA.

Control of Treg functional diversity in peripheral tissues is a promising strategy for a broad range of autoimmune diseases, cancers, and infectious diseases. Given that Nrp1 expression was negatively and strongly correlated with CD39 expression and that CD39 expression was positively correlated with CD25, CTLA-4, and GzmB expression levels (Figure 2C), regulation of Nrp1^+^ and CD39^+^ Treg migration could be used to modulate the skin inflammatory response. To provide proof of principle for this strategy, we first screened migration- and adhesion-related genes with expression levels positively correlated with *Nrp1* and *Entpd1* (encoding CD39) using scqPCR data (Figure 5A). Hierarchical clustering analysis identified two clusters including *Nrp1* and *Entpd1*, respectively. *Nrp1, Itgb1*, and *S1pr1* were in one cluster, and *Entpd1, Cxcr6, Itgae, Ccr5* and *Il2ra* in the other. We also confirmed this correlation by FCM (Figure 5B). Expression levels of CCR5, CXCR6, and CD103 (encoded by *Itgae*) were negatively correlated with Nrp1 expression and positively with both CD39 and CD25 (encoded by *Il2a*) expression levels, suggesting that regulation of these three molecules or associated signaling pathways can alter Treg functions mediated by CD39 and CD25. Expression of CD29 (encoded by *Itgb1*) was positively correlated with Nrp1 expression, consistent with correlation analysis of gene expression profiles (Figure 5A). We were not able to analyze CCR1, CCR2, CCR4, and CXCR3 expression levels because CCR1 and CCR2 were rarely expressed in skin Tregs, and expression levels of CCR4 and CXCR3 were decreased by the collagenase treatment used for cell isolation. Expression of integrin β7, encoded by *Itgb7*, was strongly correlated with CD103 (integrin αe) expression (correlation coefficient = 0.98, Figure S6A), suggesting αeβ7 heterodimer expression in skin Tregs.

Based on these correlation analyses, it appears that expression of CCR5, CXCR6, and CD103 is characteristic of CD25^+^ and/or CD39^+^ Tregs, cells with an inherent capacity to remain in skin (Figure 4). Indeed, CCR5 and CD103 expression levels were higher in Tregs remaining in skin than in Tregs migrating to skin (Figure 5C), while CXCR6 and CD29 expression levels were equivalent in these two Treg populations. We thus performed tSNE on the expression data of five molecules, CCR5, CD103, Nrp1, CD39, and CD25 (Figures S6B,C), to better separate phenotypes of Tregs migrating to and remaining in skin compared to our previous tSNE analysis based on Nrp1, CD39, CD25, GzmB, and CTLA-4 (Figure 4E). This new tSNE generated two clusters distinctly separating Tregs remaining in skin from Tregs migrating to skin (Figure S6C). Tregs remaining in skin mainly consisted of clusters with more than middle (mid) expression levels of CD25 and CD103, and the majority of CCR5^mid/high/veryhigh^ and CD39 ^mid/high/veryhigh^ cells were included in these clusters. These data suggest that CCR5 and CD103 contribute to skin retention and the functional diversity of skin-remaining Tregs.

To test this notion, we administrated the CCR5 inhibitor DAPTA into the inflamed ear 12 h prior to cell phenotype analysis. DAPTA treatment augmented Nrp1 expression and reduced both CD39 expression and the proportion of CD39^+^ cells among skin Tregs (Figure 5D). The proportion of KikGR-Red^+^ cells in skin Tregs was not altered by the treatment (Figure 5E), suggesting no change in migration activity. In addition, DAPTA treatment significantly decreased the proportion of CD39^+^ cells among Tregs remaining in skin, but not in Tregs migrating to skin (Figure 5F), although CD39 expression intensity was decreased by the treatment in both Treg populations. These data suggest that inhibition of CCR5 signaling can modulate the inflammatory process by preferentially altering the numbers of CD39^+^ and Nrp1^+^ Tregs remaining in skin (Figure 5C).

## Discussion

In this study, we demonstrate high phenotypic diversity of Tregs associated with distinct migration behaviors in the inflamed skin of CHS model mice. Several recent studies on the single-cell heterogeneity of human Tregs (10, 30, 31) have proposed multiple Treg subpopulations based on unsupervised clustering analysis of well-known surface markers for disease-associated subsets, discriminated Tregs from other CD4^+^ helper T cell subsets, or simply shown a great degree of phenotypic complexity among Tregs. However, no functional analyses were performed, largely because it is still not possible to delete specific subsets. Thus, these studies provide little information about contribution of the subsets to tissue-specific immunoinhibitory function. In the current study, to identify Treg functional diversity, we examined the single-cell expression profiles of 12 molecules, each of which has been implicated in immunoinhibitory function (32), although there are species-specific differences in suppressive effects by the molecules between human and rodents (32). Our analyses revealed a striking diversity of functional capacities among single Tregs in inflamed skin and dLNs and provided clues to immunosuppressive mechanisms.

By profiling Treg subsets at single-cell resolution, we identified multiple inter-relationships among Treg subsets expressing these functional molecules. Our findings suggest that *Nrp1* expression is likely evoked by a signaling pathway distinct from those modulating expression of the other measured genes because all expression levels except those of *Prf1* and *Nrp1* were positively correlated. Nrp1 contributes to the stabilization of Treg contact with dendritic cells (27), promotes Treg survival, homeostasis, and function (33), and is a putative marker of thymus-derived Tregs (34). Analysis by tSNE identified three distinct Treg subsets strongly expressing CD25, GzmB, or CTLA-4 and all demonstrating strong expression of CD39, supporting the associations revealed by correlation analysis. Therefore, CD39 may be a useful indicator of these three distinct subsets, which are likely to possess strong immunosuppressive functions. These data also raise the possibility of distinct activation pathways for inducing potent expression of Nrp1, CD25, GzmB, and CTLA-4 in Tregs.

The results of our correlation study established molecular targets for further investigations on the mechanisms responsible for the phenotypic and functional diversity of Tregs. We are now examining if expression levels of positively correlated gene sets are induced by one or a few common transcription factors. So, far, Ets1 has been identified by pathway analysis and other molecular experiments as a candidate transcriptional factor for induction of CD39, GzmB, and other genes correlated with *Entpd1* and *Gzmb* expression. Supporting this hypothesis, Ets1 deficiency reduced suppressive activity in Tregs (35). The signaling pathways generating Treg functional diversity are also currently under investigation.

To identify the molecular mechanism underlying T cell tissue tropism and tissue-specific function, other groups have compared single-cell expression heterogeneity of T cells, including Tregs, in lymphoid tissues to that in peripheral tissues, such as skin, colon, and lung (36, 37). However, we think that these analyses are insufficient to provide a detailed picture of tissue tropism and tissue-specific function because previous studies of the mouse lines expressing a photoconvertible protein have found that many T cells rapidly emigrate from the inflamed or non-inflamed peripheral tissues to dLNs and other lymphoid tissues (11, 21, 38, 39). These findings imply that a substantial proportion of T cells enter and exit peripheral tissues and exert transient effector functions without adaptation to the tissue environment. In a follow-up to our previous report showing the superior immunosuppressive effects of a Treg subset composed of many skin-remaining cells (11), we proposed that Tregs remaining in skin may play a central role in recovery of immune homeostasis and possess distinctive functional capacities specialized for local immunosuppression. For proof of principle, in this study we identified characteristic subsets of Tregs remaining in, migrating to, and emigrating from the inflamed skin by tSNE analysis using data without KikGR expression profiles, suggesting that skin Tregs are composed of several subpopulations with migration behavior-specific rather than tissue-specific functions. To our knowledge, this is the first report showing the unique functional phenotypic diversity of Treg subsets defined by migration behavior and suggests complex subset-specific immunoregulation in peripheral tissues.

Our data comparing Tregs remaining in skin with Tregs migrating to skin using tSNE and Phenograph analyses provide detailed information on the migration-associated functional phenotypic diversity of Tregs. Two clusters of Tregs remaining in skin exhibited very low expression of CTLA-4 and GzmB, and moderate expression of CD39 and CD25. Signaling pathways involving CD39 and CD25 alter the metabolic environment in inflamed tissue by adenosine generation and IL-2 binding, thereby providing an anti-inflammatory milieu. Alternatively, immunosuppression driven by CTLA-4 and GzmB is mediated by cell–cell contact (4, 40) and CTLA-4 expression by Tregs remaining in the skin may be unnecessary for effective local immunosuppression as suggested by results indicating that CTLA-4 serves to dampen the activation of dendritic cells in lymphoid tissue (15).

Although our ultimate goal is to comprehensively understand immune surveillance by T cells between peripheral tissues and dLN, the major limitation of the current study is the low numbers of skin Tregs and the current impossibility of isolating and testing specific Treg subsets identified by Phenograph analysis. Further, the complex dynamics of migration complicate assessment of subset differentiation and destiny. Our preliminary time course study revealed that the proportion of Tregs remaining in skin and the total number of skin Tregs remained relatively stable between 2 and 4 days following CHS induction. Hence, it follows that about half of all skin Tregs were replaced within a half day, presumably through apoptosis or emigration to dLNs. If Tregs remaining in skin also proliferate, more than half of skin Tregs could undergo apoptosis or emigrate in this period. Conversely, half of skin Tregs are migrating from dLNs or other tissues to the inflamed skin. However, there is currently no method available for measuring the influx rate of cells destined to remain in the skin. We are also unable to test whether some Tregs have an inherent capacity to remain in skin or whether and how they acquire this capacity. A related question is whether skin Tregs strongly express CD39 and/or CD25 before or after recruitment to the skin. To resolve these questions, comprehensive spatiotemporal studies simultaneously measuring cellular migration, death, and proliferation are required. We are currently developing mouse lines to facilitate such studies.

The positive correlations of CD39 and CD25 expression levels with CCR5 expression suggest that CCR5 signaling contributes to CD39^+^ and CD25^+^ Treg residence in inflamed skin and percutaneous treatment with a CCR5 inhibitor changed the functional diversity of skin Tregs by altering their migration behavior. CCR5 signal inhibition altered CD39 and Nrp1 expression levels in skin Tregs, suggesting that this chemokine signaling pathway is associated with Treg functional diversity. Although CCR5 signal inhibition did not reduce the proportion of KikGR-Red^+^ cells among skin Tregs, we observed that Tregs remaining in the skin were preferentially influenced by this treatment compared to Tregs migrating to skin. In addition to CCR5, CCR4 is a well-known chemokine receptor expressed in Tregs (13); however, Treg depletion by anti-CCR4 antibody has not translated into therapeutic efficiency against cancer (41). Therefore, new molecular targets are required for Treg regulation, and our data linking migration behavior to expression profiles of specific functional molecules in Tregs may provide future targets for therapeutic modulation of Treg function.

In conclusion, these single-cell expression data combined with tracking of Treg migration identify functionally distinct subsets of Tregs in skin. Further, these results highlight important contributions by Nrp1, CD25, Granzyme B, CTLA-4, CD39, and CCR5 among other trafficking signaling molecules in conferring unique migratory and tissue-retention capacities and possibly also tissue-specific immunoinhibitory activities to Tregs.

## Ethics Statement

All animals were treated according to the Guidelines for Proper Conduct of Animal Experiments (Science Council of Japan), and all protocols were approved by the Institutional Animal Care and Use Committee of Kyoto University Faculty of Medicine and the Animal Research Committee of Osaka Ohtani University.

## Author Contributions

RI and MT: designed experiments. RI and MF: performed experiments. RI: performed statistical analysis and data visualization. RI, YN, HO, TM, YK, and MT: analyzed data. RI and MT: wrote the manuscript.

### Conflict of Interest Statement

The authors declare that the research was conducted in the absence of any commercial or financial relationships that could be construed as a potential conflict of interest.
