# Supplementary material for: Functional Phenotypic Diversity of Regulatory T Cells Remaining in Inflamed Skin
Source: Front Immunol. 2019 May 17;10:1098. doi: 10.3389/fimmu.2019.01098 (PMC6534040; doi:10.3389/fimmu.2019.01098)
Supplement: Supplementary file 1 [file Data_Sheet_1.pdf]

Supplementary information for

Functional phenotypic diversity of regulatory T cells remaining in inflamed skin

Ryoyo Ikebuchi, Maika Fujimoto, Yasutaka Nakanishi, Hiromi Okuyama, Taiki Moriya, Yutaka Kusumoto, Michio Tomura

Figure S1. Gene expression profiles for all genes examined

Figure S2. Cross-correlation analysis using FCM data with two sets of multicolor panels

Figure S3. tSNE analysis of skin and dLN Tregs with different migration activity

Figure S4. tSNE analysis of Tregs migrating to and remaining in skin

Figure S5. Clustering analyses of skin Tregs

Figure S6. tSNE analysis using expression profiles of CCR5, CD103, Nrp1, CD39, and CD25 in skin Tregs

Table S1. Sequences of antibodies using for sorting and flow cytometer

Table S2. Sequences of primers using for pre-amplification and scqPCR

Table S3. Cell numbers of Tregs with different migration status in clusters generated by manual gating

A

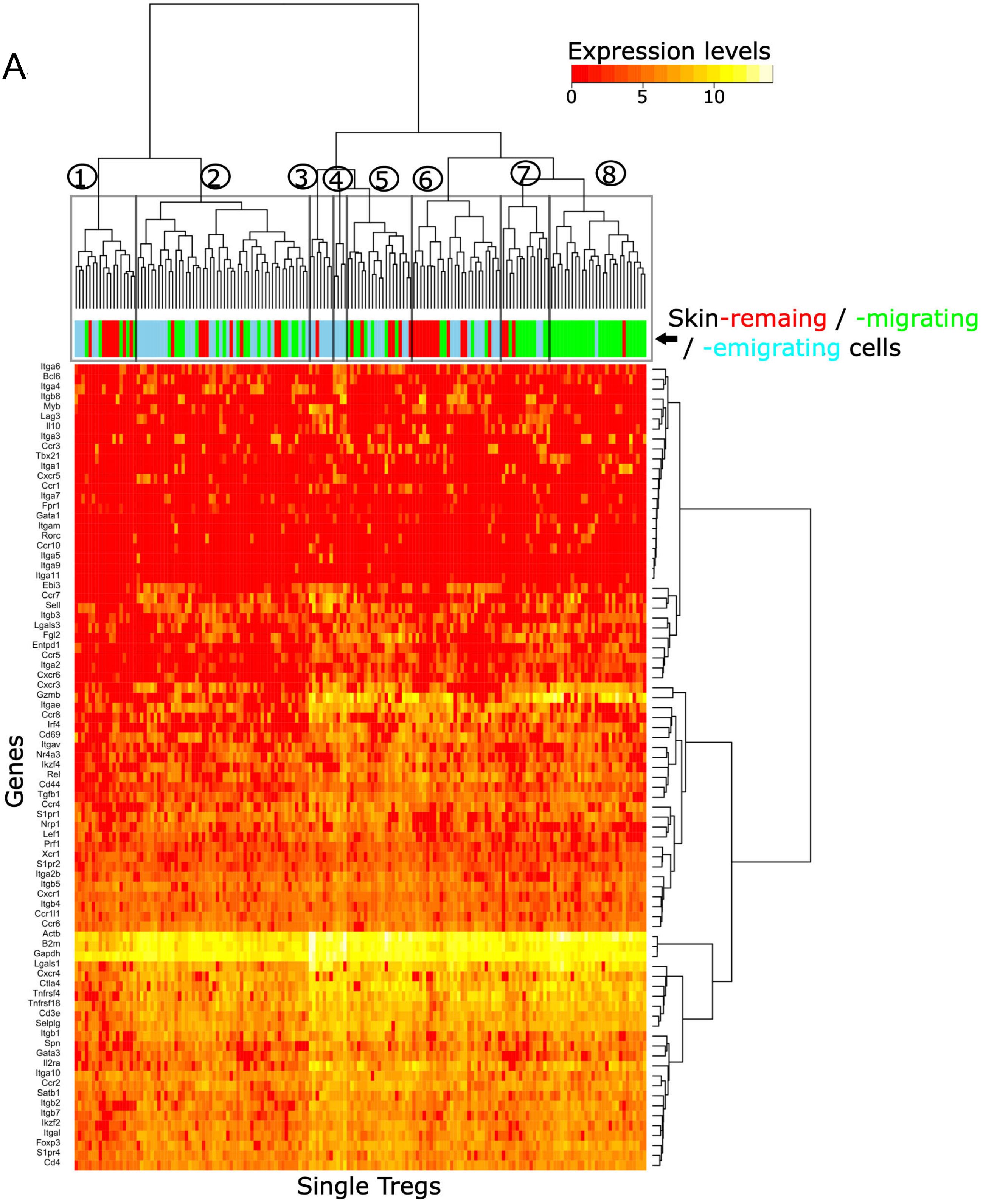
Expression levels
0
5
10
1
2
3
4
5
6
7
8
Skin-remaing / -migrating / -emigrating cells
Genes
Itga6
Bcl6
Itga4
Itgb8
Myb
Lag3
Il10
Itga3
Ccr3
Tbx21
Itga1
Cxcr5
Ccr1
Itga7
Fpr1
Gata1
Itgam
Rorc
Ccr10
Itga5
Itga9
Itga11
Ebi3
Ccr7
Sell
Itgb3
Lgals3
Fgl2
Entpd1
Ccr5
Itga2
Cxcr6
Cxcr3
Gzmb
Itgae
Ccr8
Irf4
Cd69
Itgav
Nr4a3
Ikzf4
Rel
Cd44
Tgfb1
Ccr4
S1pr1
Nrp1
Lef1
Prf1
Xcr1
S1pr2
Itga2b
Itgb5
Cxcr1
Itgb4
Ccr1l1
Ccr6
Actb
B2m
Gapdh
Lgals1
Cxcr4
Ctla4
Tnfrsf4
Tnfrsf18
Cd3e
Selplg
Itgb1
Spn
Gata3
Il2ra
Itga10
Ccr2
Satb1
Itgb2
Itgb7
Ikzf2
Itgal
Foxp3
S1pr4
Cd4
Single Tregs

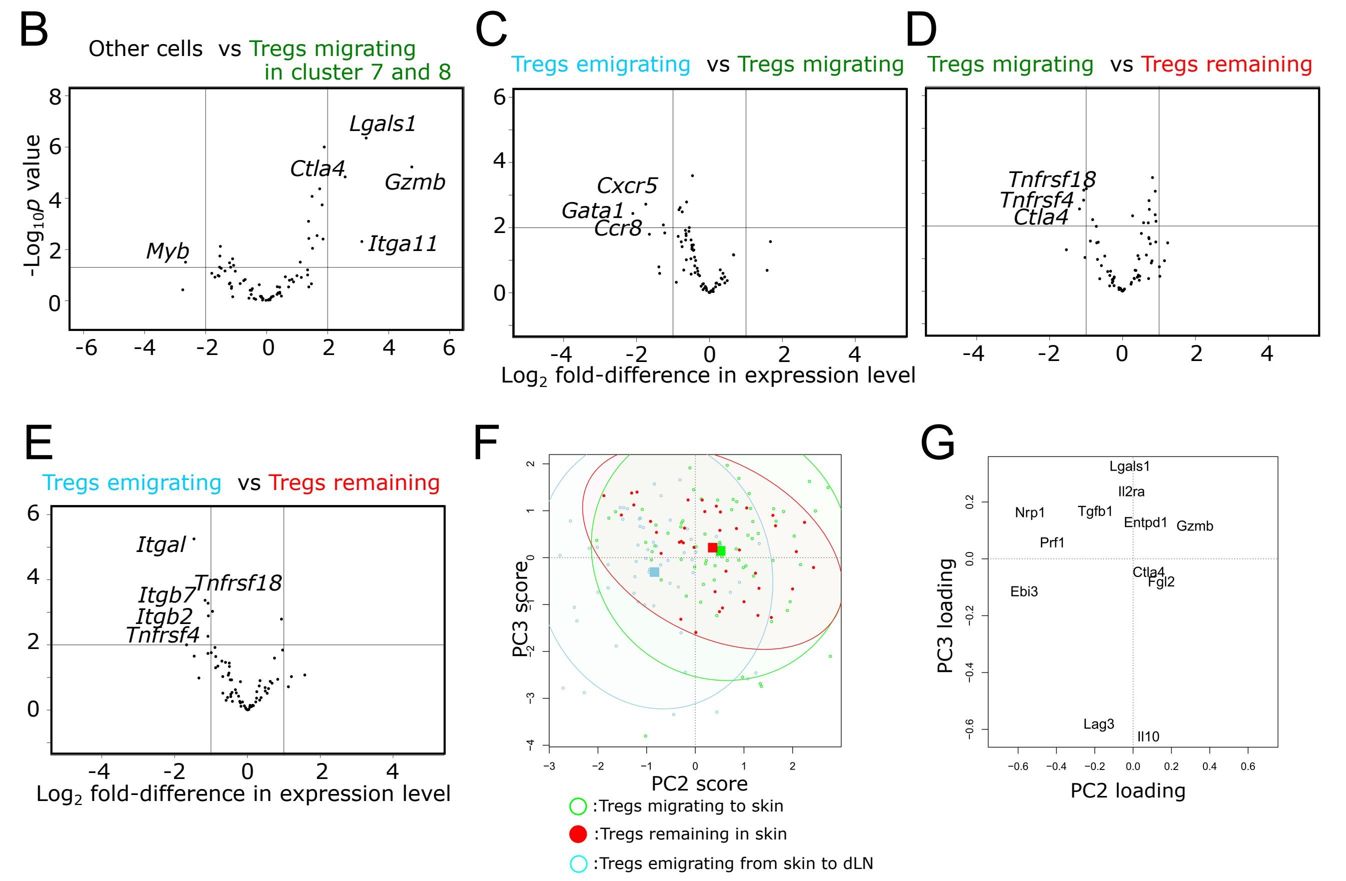

Figure S1. Gene expression profiles for all genes examined
(A) Heatmap showing all single-cell expression data. Migration activities of cells are depicted as follows: Tregs migrating to skin (green), Tregs remaining in skin (red), Tregs emigrating from skin to dLN (cyan). Numbers in a tree diagram of single cells indicate cluster numbers. Clustering was performed by the Manhattan distance and Ward's method. (B, C, D, and E) Volcano plots indicating genes differentially expressed in Tregs migrating to skin in clusters 7 and 8 and between each of the three Treg populations. Vertical lines indicate a two-fold change in expression. Horizontal lines indicate p = 0.01. Selected genes showing statistically significant differences in expression among populations labeled. (F) PC projections of individual Tregs within the three migratory populations. PC2 and PC3 are shown (accounting for 26.5% and 12.5% of the total variation, respectively). The confidence ellipses help to visually compare relationships among the populations. Squares indicate the mean value for each subset with the same color. (G) PC projection of 12 genes, showing contributions to PC2 and PC3.

A

Fluorochrome	Molecule
PE-Cy7	CD39
Alexa Fluor 647	GzmB
PE-Dazzle594	CTLA-4
BV711	CD25
BV421	Nrp1

B

Fluorochrome	Molecule
PE	CD39
Pacific Blue	GzmB
APC	CTLA-4
PE-Dazzle594	CD25
PE-Cy7	Nrp1

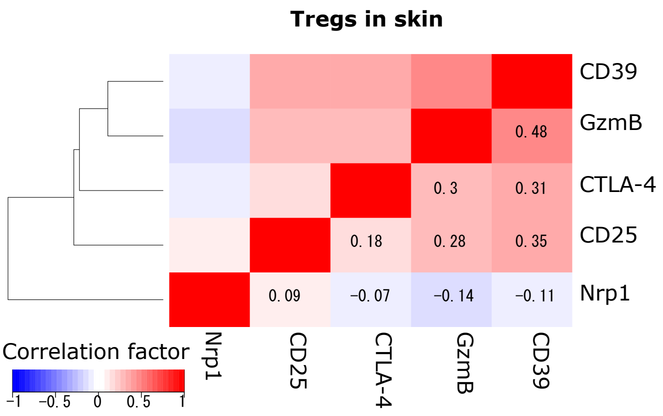

Figure S2. Cross-correlation analysis using FCM data with two sets of multicolor panels
(A) FCM multicolor panel used in Fig. 2B and C. (B) Cross-correlation analysis of the expression profiles of five proteins using pooled FCM data with a different color panel from (A) (*n* = 3). Clustering was performed by the Manhattan distance and the group average method.

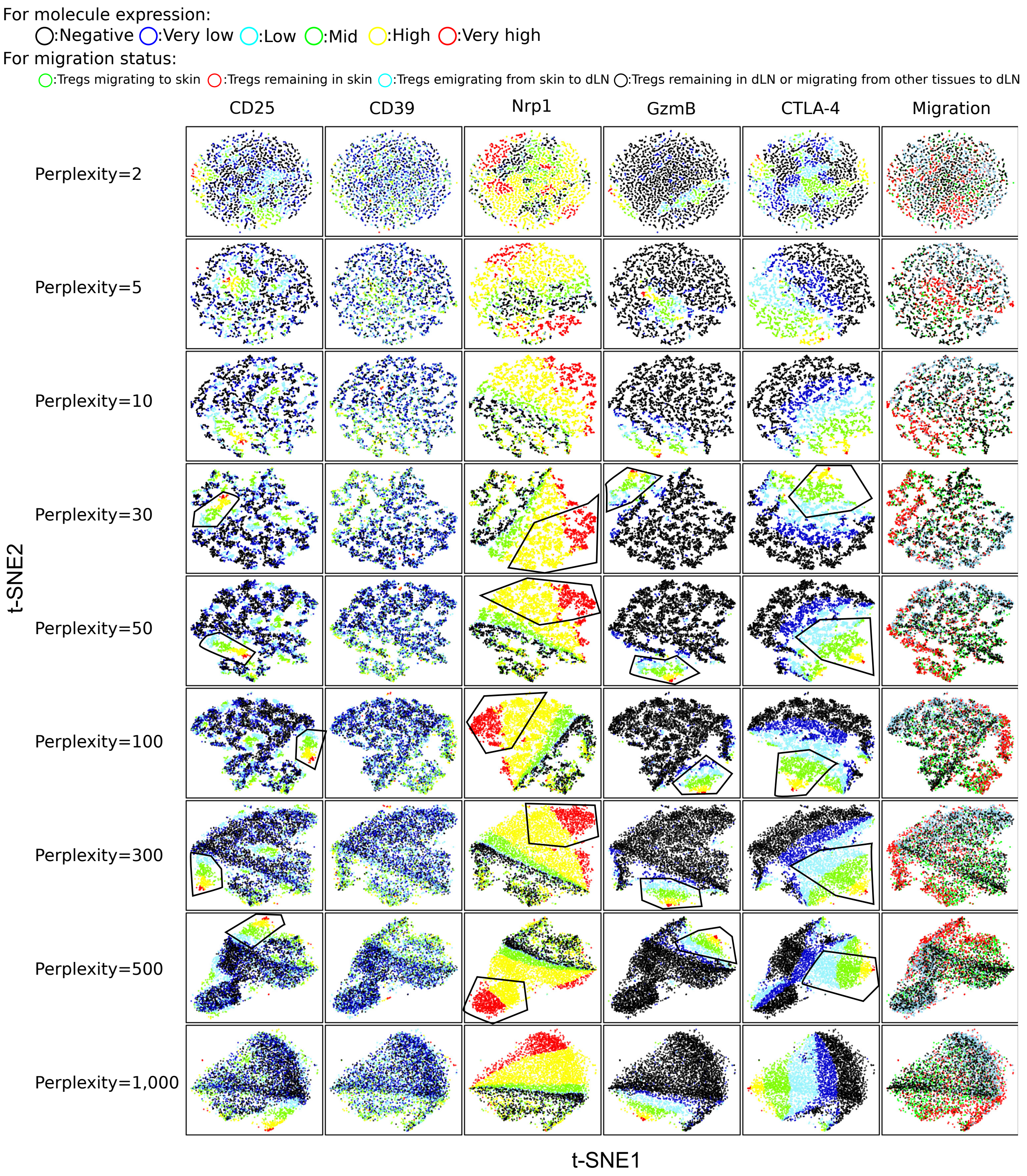

Figure S3. tSNE analysis of skin and dLN Tregs with different migration activity
tSNE projections of different perplexities using same data of Fig. 3. Six expression levels (defined as in Fig. 3A) are indicated by different colors. Clusters named CL-25, CL-Nr, CL-Gz, and CL-CT were indicated in tSNE plots showing expression of CD25, Nrp1, GzmB, and CTLA-4 (polygons). Migration activities of individual cells are indicated in the tSNE plots to the extreme right (migration) by different colors.

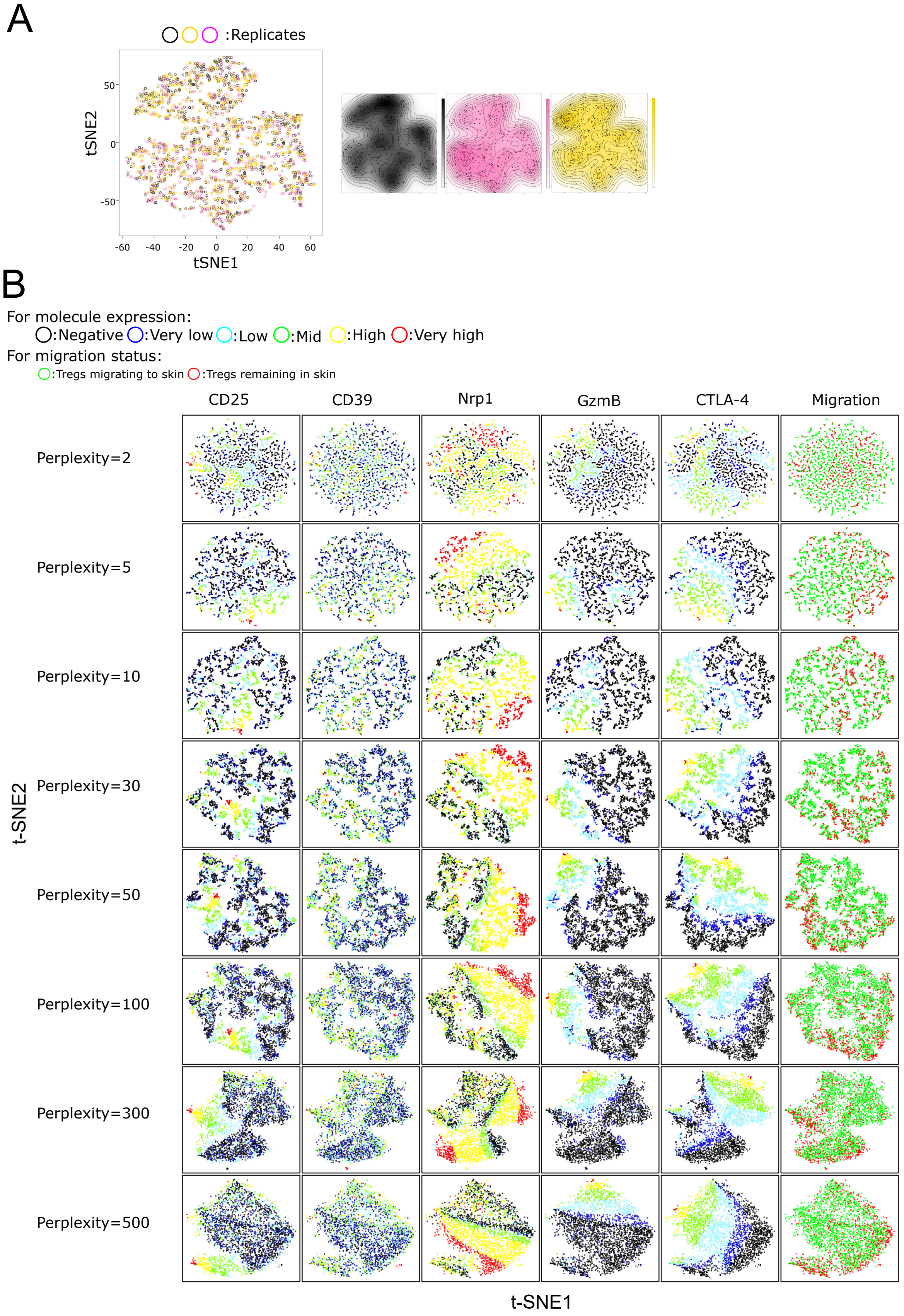

Figure S4. tSNE analysis of Tregs migrating to and remaining in skin

(A) tSNE projection of skin Tregs. Cells in each triplicate datasets are indicated by a different color. (B) tSNE projections of different perplexities using same data of Fig. 4. Expression levels and migration activity of individual cells are indicated as in Fig. S3.

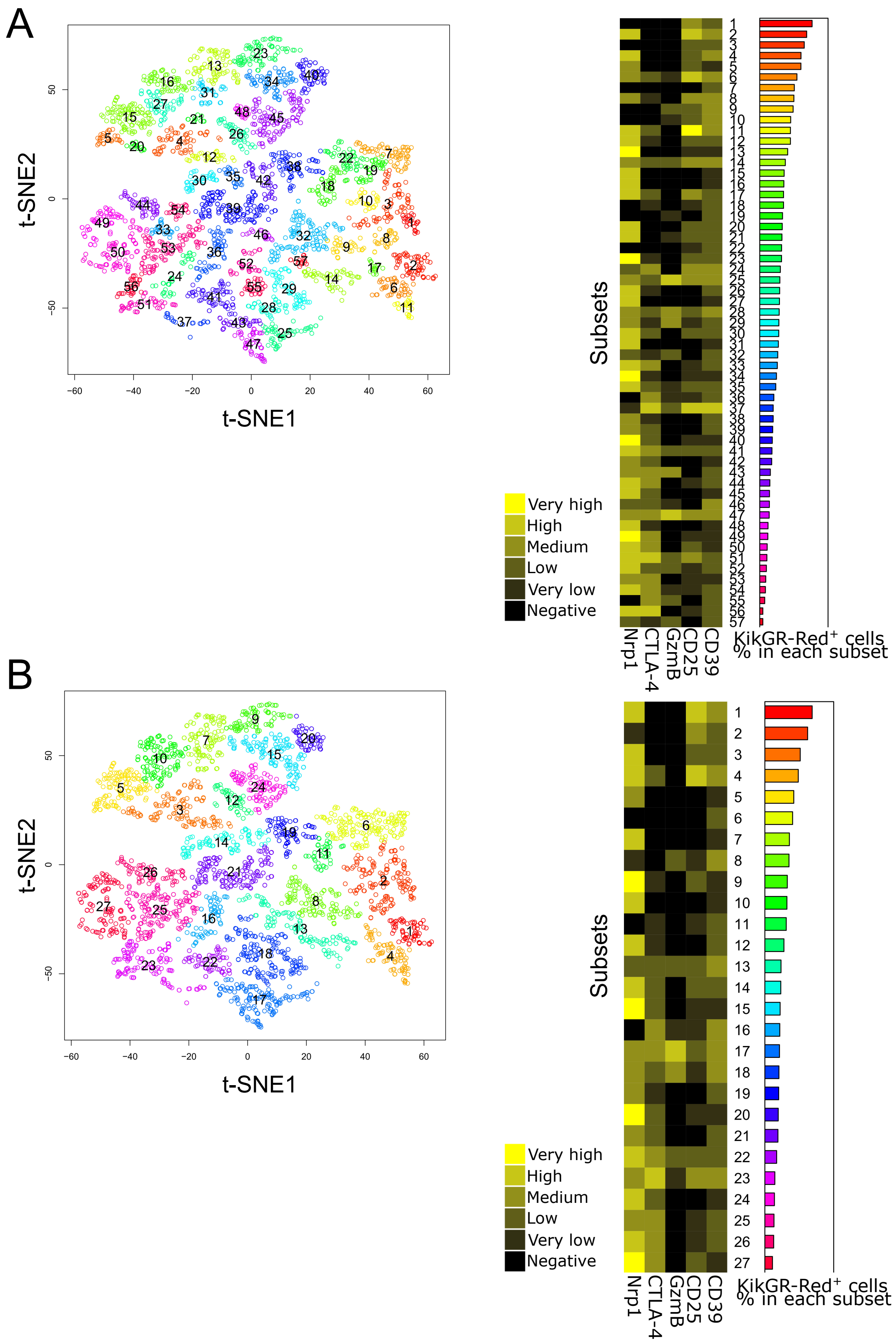

Figure S5. Clustering analyses of skin Tregs

(A, B) Mean expression of the five molecules in each of 57 and 27 subsets identified by Phenograph using data of tSNEV1 and V2 (k=12 and 30).

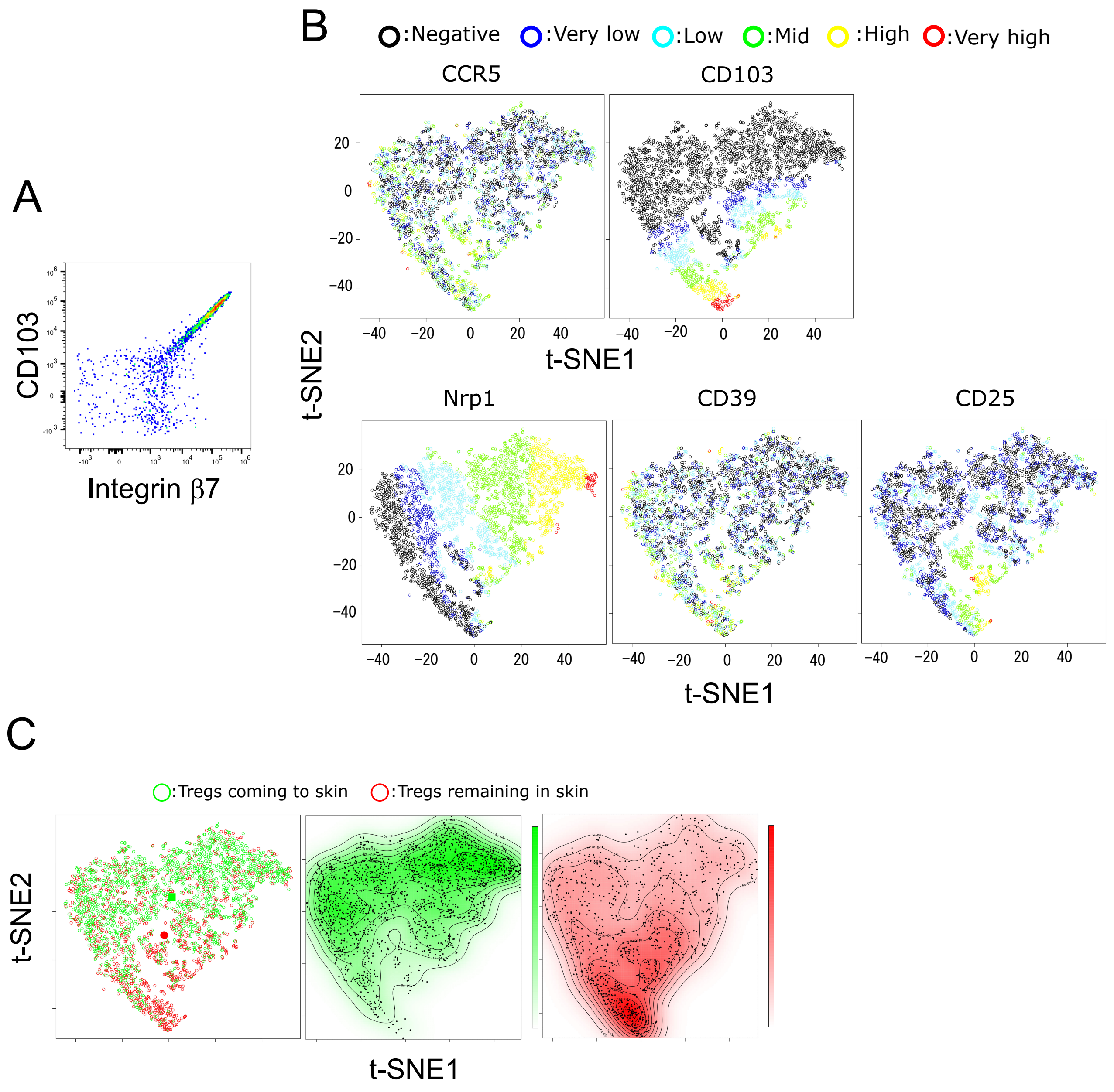

Figure S6. tSNE analysis using expression profiles of CCR5, CD103, Nrp1, CD39, and CD25 in skin Tregs

(A) Representative data of CD103 (Integrin αE) and Integrin β7 expression in skin Tregs (n = 2). (B, C) tSNE projections of seven proteins expressed by Tregs from pooled data of individual Tregs migrating to and remaining in skin (*n* = 3). Six expression levels (defined as in Fig. 3A) and the migration activity of individual cells are indicated by different colors. Clusters were defined by manual gating (polygon).

Table S1. Sequences of antibodies using for sorting and flow cytometer

Molecules	Conjugation	Manufacturers
Human CD2	BV421<br>Pacific blue<br>BV510	Biolegend<br>Biolegend<br>Biolegend
CD4	APC<br>BV785<br>Alexa Fluor 700	BD<br>Biolegend<br>Biolegend
CD25	BV711<br>PE-Dazzle594	Biolegend<br>Biolegend
CD29	PE-Cy5	Biolegend
CD39	PE-Cy7<br>PE	Biolegend<br>Biolegend
CD45	eFluor650<br>BV510	eBioscience<br>Biolegend
CD45.2	PE-Cy7<br>APC-Alexa Fluor 750	Biolegend<br>eBioscience
CD103	PE-Dazzle594	Biolegend
CD186 (CXCR6)	PE	Biolegend
CD195 (CCR5)	Biotin	Biolegend
CD223 (LAG3)	Biotin	Biolegend
CD152 (CTLA-4)	PE-Dazzle594<br>APC	Biolegend<br>Biolegend
CD304 (Nrp1)	PE<br>BV421<br>PE-Cy7	Biolegend<br>Biolegend<br>Biolegend
GzmB	Alexa Fluor 647<br>Pacific blue	Biolegend<br>Biolegend

Table S2. Sequences of primers using for pre-amplification and scqPCR

Gene Symbol	Forward primer	Reverse primer	Nested forward primer	Nested reverse primer
Actb	TGACAGGATGCAGAAGGAGA	CGCTCAGGAGGAGCAATG	CAGGATGCAGAAGGAGATTACTG	GGAGGAGCAATGATCTTGATC
B2m	ACTGACCGGCCTGTATGCTA	ATGTTCGGCTTCCCATTCTCC		
Bcl6	CTGCAGATGGAGCATGTTGT	GCCATTTCTGCTTCACTGG		
C5ar1	GCATCCGTCGCTGGTTAC	TGCTGTTATCTATGGGGTCCA		
Ccr1	TCCTCAAAGGCCCAGAAACA	GCTGAGGAACTGGTCAGGAA		
Ccr10	TAGCCAGAGATGGGGACCAA	CGAATAGGCCTCCTCATCGTAC		
Ccr1l1	CCTCCTCTTGGCCCCTTAT	GGGTGAATAGGACGTCTTCAAA		
Ccr2	TGAGGCTCATCTTTGCCATCA	GGATTCCTGGAAGGTGGTCAA		
Ccr3	CTGGACTCATAAAGGACTTAGCA	GTGGTGCCCACTCATATTCA		
Ccr4	TGTCCTCAGGATCACTTTCAGA	GGCATTCATCTTTGGAATCG		
Ccr5	TAGCCAGAGGAGGTGAGACA	CGGAACTGACCCTTGAAAATCC		
Ccr6	AAGGCACATATGCGGTCAAC	CCTGGACGATGGCAATGTAC		
Ccr7	GTGGTGGCTCTCCTTGTCA	GGTATTCTCGCCGATGTAGTCA		
Ccr8	AGTGGGCAGCTCTGAAACC	TTGGGCTCCATCGTGTAATCC		
Ccr9	CACCATGATGCCCACAGAAC	CTGTGGAAGCAGTGGAGTCATA		
Cd3e	TGCTACACACCAGCCTCAAA	AGGTCCACCTCCACACAGTA		
Cd4	AAGGGACACTGCATCAGGAA	CCCATCACCTCACAGGTCAA		
Cd44	TTCCTTCGATGGACCGGTTA	TACTCGCCCTTCTTGCTGTA		
Cd69	GTGGTCCTCATCACGTCCTTA	ACAAGCCTGGGCAATTGTAC		
Cmklr1	GCGAGTTCTCAAACCCTGAA	TGCGCGTGACTGGAAATAC		
Ctla4	GGACTTGGCCTTTTGTAGCC	CTGAAGGTTGGGTCACCTGTA		
Cx3cr1	AAGTTCCCTTCCCATCTGCT	CAAAATTCTCTAGATCCAGTTCAGG		
Cxcr1	TTCTGAGCTTGCTGGGAAAC	GGGTCCTTCGCCTGTATAAGA		
Cxcr2	CACAAACAGCGTCGTAGAACTA	AGGGCATGCCAGAGCTATAA		
Cxcr3	ACCAGCCAAGCCATGTACC	GGGAGAGGTGCTGTTTTCCA		
Cxcr4	GGTAACCACCACGGCTGTA	CAGGGTTCCTTGTTGGAGTCA		
Cxcr5	GGACATGGGCTCCATCACATA	GGGGAATCTCCGTGCTGTTA		
Cxcr6	AGCTACTGGGCTTCTCTTCTGA	TCGTAGTGCCCATCGTACAG		
Cxcr7	CACCGTCAGGAAGGCAAA	CCAGGCTCTGCATAGTCAAA		
Ebi3	GCTCCCCTGGTTACACTGAA	ACGGGATACCGAGAAGCAT	CCTGGTTACACTGAAACAGCTC	GGATACCGAGAAGCATGGC
Entpd1	GCAAGCAGAGACAGCAAAAAC	GCAAAATCTCTTCACCTTAGAATCC		
Fgl2	GCCAAGAACACATGCAGTCA	ACTCTGTAGGCCCCACTGC		
Foxp3	CCCACACCTCTTCTTCCTTGAA	GACGGTGCCACCATGACTA		
Fpr1	TGTCCAGAGCTGTTGGAAAGT	TTCATGAGGTTCACTGCAGACT		
Gapdh	AGACGGCCGCATCTTCTT	TTCACACCGACCTTCACCAT		
Gata1	GCATCAACAAGCCCAGGT	AAACTGGGGCAAGGGTTCT		
Gata3	CCTACCGGGTTCGGATGTAA	CCGCAGTTCACACACTCC	CGGGTTCGGATGTAAGTCGA	GTTCACACACTCCCTGCC
Gpr17	TCCTTTCCTTCCTGGGTCTT	CCAGGGAGGAGTTGTCAGTC		

Gzmb	GCATTCCCCACCCAGACTAT	AGTCCTCTTGGCCTTACTCTTC	TCCCCACCCAGACTATAATC	TGGCCTTACTCTTCAGCTTTA
Ikzf2	GGCCCAATGTGCTTATGGTAC	AAGAAGCTCCGCACTGGTTA		
Ikzf4	CTGATGCCCAAGCTCTGAC	AGCATTGAGTCAGGCACCA		
Il10	AAAGGACCAGCTGGACAACA	TAAGGCTTGGCAACCCAAGTA		
Il12a	TCAGAATCACAACCATCAGCA	CGCCATTATGATTCAGAGACTG		
Il2ra	TGCGTTGCTTAGGAAACTCC	CTGGTGTTCAAGTTGAGCTGTA		
Irf4	TCCCCATTGAGCCAAGCATA	CGAGGATGTCCCGGTAATACA		
Itga1	GATGGGGACGTCAACATTCT	TGTGGTTAAGACGCTACCAAAG		
Itga10	TGCTTCAAGCTGACATGGAC	TCCACCTTGAACCACAAATG		
Itga11	GCAGACGTCCTCTTTACCAGA	GAGCTGTTTGCCTTGACCTC		
Itga2	ACTTCCGGCATACGAAAGAA	TCAGCCAGCAGGTGATGTTA		
Itga2b	AAGCTGAAGCCACAGTGGAG	TGGAGACCCATCTGTCCAA		
Itga3	TCAACATGGAGAACAAGACCA	CCAACCACAGCTCAATCTCA		
Itga4	ACTCCCCACAGGCCTTTATT	TCAGTCACTTCGCAGTTTATTTG		
Itga5	CACCATTCAATTTGACAGCAA	TCCTCTCCCTTGGCACTGTA		
Itga6	GTATTCAGGAGTAGCTTGGTGGA	TTTCTCTTGAAGAAGCCACACTT		
Itga7	AAAACTTTGGACCCTGCTGA	CCAGAATCGATGGAGAAACC		
Itga8	ATCACTGCCATTGCTCAGGT	GTGGATGGGCAGAACAATCT		
Itga9	CAACAGAGAGGGAGCGTTCT	CCTCACCCATCACATGCTC		
Itgad	ACATGCCATGGTGGGTCT	GCCCCATTCTTGCTATGAAA		
Itgae	CTCAGGTGGCTTGTATGACAGT	CAGACATGCCGAAGTAGTGG		
Itgal	CCCCAGACTTTTGCTACTGG	TAGGCCGTGTGTCCAGGT		
Itgam	CAATAGCCAGCCTCAGTGC	GAGCCCAGGGGAGAAGTG	TAGCCAGCCTCAGTGCCT	GGAGAAGTGGGCGCC
Itgav	GGTGTGGATCGAGCTGTCTT	CAAGGCCAGCATTTACAGTG		
Itgax	TCGTATTTGGCTTCCCAGAC	CCATCATTAGACACCGTCACAT		
Itgb1	CAACCACAACAGCTGCTTCTAA	TCAGCCCTCTTGAATTTTAATGT		
Itgb2	CCCAGTGTGAGTGTCAGTGC	TCCCAATGTAGCCAGACTCA		
Itgb3	GTGGGAGGGCAGTCCTCTA	CAGGATATCAGGACCCTTGG		
Itgb4	GCAGGAGCCACAGTGTGAT	CCGCCGTTTAGTAGCTGGT		
Itgb5	CGTTGTGTCTGTGGGCAGT	GCAAGCATTCGACACAGTCT		
Itgb6	TCTAAGGCCAAGTGGCAAAC	TGCTTCTCCCTGTGCTTGTA		
Itgb7	TGTGCATGGTGCAAACAAC	GCGAGCCAGTAGCTCCTCT		
Itgb8	ACTTCTCCTGTCCCTATCTCCA	ATCTGCCACCTTCACACTCC		
Lag3	CACCTGTAGCATCCATCTGC	CCAGGTAACCCGAAGGATTT		
Lef1	TCCTGAAATCCCCACCTTCT	TGGGATAAACAGGCTGACCT		
Lgals1	CTCAAAGTTCGGGGAGAGGT	CATTGAAGCGAGGATTGAAGT	GAGAGGTGGCCTCGGA	AAGCGAGGATTGAAGTGTAGG
Lgals3	ATCATGGGCACAGTGAAACC	AGTGGAAGGCAACATCATTCC		
Myb	TGTCAACAGAGAACGAGCTGA	GCTGCAAGTGTGGTTCTGTG	AACAGAGAACGAGCTGAAGGG	TGCAAGTGTGGTTCTGTGTT
Nr4a3	CTGCCTGTCAGCACTGAGTA	CTTGGTGCATAGCTCCTCCA		
Nrp1	TCCTGGGAAACTGGTATATCTATGA	CATTCCAGAGCAAGGATAATCTG		
Prf1	GAAGAAGAAACAGCACAAAATGG	GACGTGACGCTCACGGTAG		

Rel	TTGCAGAGATGGATACTATGAAGC	CACCGAATACCCAAATTTTGAA		
Rorc	TGGAGCTCTGCCAGAATGAC	GGCCCTGCACATTCTGACTA		
S1pr1	CGGTGTAGACCCAGAGTCCT	AGCTTTTCCTTGGCTGGAG	TGTAGACCCAGAGTCCTGCG	CTTTTCCTTGGCTGGAGAG
S1pr2	CCCAACTCCGGGACATAGA	ACAGCCAGTGGTTGGTTTTG		
S1pr3	GCCCCTAGACGGGAGTCTTA	ACTGCGGGAAGAGTGTTGAAA		
S1pr4	GGTGTACTACTGCCTGCTGAA	CTGACAGCAGCACGTTGAC		
S1pr5	GGAGTGCCGGTTACAGGAG	GAAGGACAATAACCTCACTCACC		
Satb1	AGTGATCCGAAGGGTCCAC	CCCAAGCCTTCCTCTTCCTA		
Sell	TGGTCATCTCCAGAGCCAAT	GCAGTCCATGGTACCCAACT		
Selplg	AGGCAGAGTCGTTTGCTTCT	ACAAGGAAGCTTGGGGACAT		
Spn	GCCCTGTGCCTTAACCATT	GAAGGTGCAAGGCCATCTC	CCTTAACCATTAATCTGGTCCC	GTGCAAGGCCATCTCCA
Tbx21	CAACCAGCACCAGACAGAGA	ACAAACATCCTGTAATGGCTTG		
Tgfb1	GCTGCGCTTGCAGAGATTAA	GTAACGCCAGGAATTGTTGCTA		
Tnfrsf18	GACGGTCACTGCAGACTTTG	TCCTCCTCAGGGAACTGGA		
Tnfrsf4	GCTTGGAGTTGACTGTGTTCC	GGGTCTGCTTTCCAGATAAGG		
Tnfrsf9	TTCTCTCCCAGTACCACCATTT	GAGCTGCTCCAGTGGTCTTC		
Xcr1	ACATGATACCCATGGGGAAGT	GTGCACGAAGTGTTGCTTTG		

Nested primers were used to amplify the genes, *Actb*, *Ebi3*, *Gata3*, *Gzmb*, *Itgam*, *Lgals1*, *Myb*, *S1pr1* and *Spn* in real-time PCR using Biomark.

Table S3. Cell numbers of Tregs with different migration status in clusters generated by manual gating

(Cells)	Tregs coming to skin	Tregs remaining in skin	Tregs emigrating from skin to dLN	Tregs remaining in dLN or migrated from other tissues to dLN	Total
**CL-Nr**	253	239	645	430	1,567
**CL-Gz**	268	218	33	54	573
**CL-25**	75	259	48	23	405
**CL-Ot**	246	300	311	267	1,124
**CL-CT**	351	177	156	419	1,103
**Total**	1,193	1,193	1,193	1,193	4,772
